# Development of ESAT-6 Based Immunosensor for the Detection of *Mycobacterium tuberculosis*

**DOI:** 10.3389/fimmu.2021.653853

**Published:** 2021-05-19

**Authors:** Rishabh Anand Omar, Nishith Verma, Pankaj Kumar Arora

**Affiliations:** ^1^ Department of Environmental Microbiology, Babasaheb Bhimrao Ambedkar University, Lucknow, India; ^2^ Centre for Environmental Science and Engineering, Indian Institute of Technology Kanpur, Kanpur, India; ^3^ Department of Chemical Engineering, Indian Institute of Technology Kanpur, Kanpur, India

**Keywords:** *Mycobacterium tuberculosis*, cyclic voltammetry, ESAT-6, immunosensor, reduced graphene oxide, polyaniline

## Abstract

Early secreted antigenic target of 6 kDa (ESAT-6) has recently been identified as a biomarker for the rapid diagnosis of tuberculosis. We propose a stable and reusable immunosensor for the early diagnosis of tuberculosis based on the detection and quantification of ESAT-6 *via* cyclic voltammetry (CV). The immunosensor was synthesized by polymerizing aniline dispersed with the reduced graphene oxide (rGO) and Ni nanoparticles, followed by surface modification of the electroconductive polyaniline (PANI) film with anti-ESAT-6 antibody. Physicochemical characterization of the prepared materials was performed by several analytical techniques, including FE-SEM, EDX, XRD, FT-IR, Raman, TGA, TPR, and BET surface area analysis. The antibody-modified Ni-rGO-PANI electrode exhibited an approximately linear response (R^2^ = 0.988) towards ESAT-6 during CV measurements over the potential range of -1 to +1 V. The lower detection limit for ESAT-6 was approximately 1.0 ng mL^-1^. The novelty of this study includes the development of the reusable Ni-rGO-PANI-based electrochemical immunosensor for the early diagnosis of tuberculosis. Furthermore, this study successfully demonstrates that electro-conductive PANI may be used as a polymeric substrate for Ni nanoparticles and rGO.

## Introduction

Tuberculosis (TB) is an airborne disease that can be transmitted through coughing, sneezing, laughing, and even talking (1). It is one of the leading causes of death in the world. The causative agent of tuberculosis is the *Mycobacterium tuberculosis* (*Mtb*) bacterium. Therefore, an early detection of *Mtb* is critical to preventing the spread of infection and to eradicating the disease.

Many traditional biochemical methods, including acid-fast staining, culturing, and colony counting have been used to detect tuberculosis. However, these methods are time-consuming, often inaccurate, and provide only qualitative data. In recent years, various transduction techniques have been developed using fiber optics, surface plasmon resonance, piezoelastics, and magnetoelastics. These techniques are rapid and accurate but too expensive to be used on a diagnostic level, especially in developing countries where the spread of *Mtb* is common (2–4).

In recent years, the development of electrochemical sensors to diagnose *Mtb* has drawn keen interest. Electrochemical sensors identify a biomarker using a suitable recognition element that is immobilized on a substrate. A change in the current response occurs when the recognition element interacts with biomarkers in the diagnostic fluid. In this context, poly L-lysine (5), antigen-specific antibodies (6–8), and DNA aptamers (9–12) have been successfully tested as the recognition elements. Although such sensors are accurate and fast, extraction of biomolecules (including DNA) from clinical samples is tedious and complex, requiring a sophisticated molecular laboratory to process samples collected from patients infected with tuberculosis. The preparation cost is also high, considering that DNA probes must be specifically grafted to the substrate (13–15).

Early secreted antigenic target of 6 kDa protein (ESAT-6) is the major virulence factor of *Mycobacterium*, which is secreted in the blood and sputum of infected persons. ESAT-6 is secreted only by the *Mycobacterium* pathogenic species. Thus, it is a potential biomarker for *Mtb*, whose detection can be rapid, specific, and accurate at all stages of the infection. It should be noted that the *Mtb*-infected sputum and blood also contain other proteins such as CPF-10 and antigen-85, but monoclonal antibodies are specific to their antigens, binding only through a specific epitope (16). A gold-plated screen-printed electrode (SPE) has been successfully immobilized with the anti ESAT-6 antibody as the recognition element for the *Mtb* biomarker at 7 ng mL^-1^ (17). The electrochemical sensor showed a good linearity (0.992) over the measured concentration range. However, the sensor was not tested for stability and reusability. Moreover, SPE is expensive, making production of the sensors cost-prohibitive.

Recent studies have demonstrated that the metal-reduced graphene oxide (rGO) composite-based sensors are capable of detecting various biomolecules such as cholesterol, creatinine, and glucose (18–21). Graphene-based materials also have some unique physicochemical properties such as adsorption, chemical stability, and amenability to surface functionalization, which can facilitate detection of a wide range of biomolecules (22, 23). Inclusion of the metal nanoparticles (NPs) such as Au, Ag, Cu, Co, and Ni in the electrode material increases the sensitivity and speed of the sensor, attributed to an increased direct electron transfer (24–32). However, Au and Ag are expensive metals. While Cu and Co are inexpensive, they display cytotoxicity and have unstable (transient) oxidation states. Ni is an inexpensive, non-toxic, and stable metal used for sensing applications. Further, polyaniline (PANI) is frequently used as a conductive material for many electrical and electronic applications (20, 33). Therefore, a composite of Ni, rGO, and PANI is a potential candidate for the electrode, and is the focus of the present study.

Here, we describe a cyclic voltammetry (CV)-based immunosensor using a Ni-rGO-PANI electrode that targets the ESAT-6 virulence factor of *Mtb* using the anti-ESAT-6 antibody as the recognition element. The developed sensor is capable of detecting ESAT-6 both qualitatively and quantitatively. To the authors’ knowledge, this is the first study that describes the Ni-rGO-PANI-based electrochemical immunosensor for the detection of *Mtb* infection at an early stage. Furthermore, the electroconductive PANI is used as the polymeric substrate for the Ni and rGO NPs, also for the first time. The prepared sensor is tested at different ESAT-6 concentrations and its performance is compared to published data, wherever possible.

## Material and Methods

### Chemicals and Reagents

ESAT-6 (Pro-291) and Ag85B (Pro-589) proteins were purchased from Prospec Protein Specialist (Germany). Anti-ESAT-6 monoclonal antibody (SC-57730) was purchased from Santa Cruz Biotechnology (Germany). Dithiobis (succinimidyl propionate) (DSP) was purchased from TCI chemicals (India). Graphite powder were purchased from S.D. Fine Chemical Ltd (India). 4-acetamidophenol (AP), glucose (Glu), uric acid (U), L-ascorbic acid (AA), creatinine (Cre), cholesterol (Chl), barbituric acid (BA), and L-glutamine (Glt) were purchased from Tata Chemicals, India. Bovine albumin serum (BSA), aniline monomer, ammonium persulfate ((NH_4_)_2_S_2_O_8_), disodium hydrogen phosphate (Na_2_HPO_4_), potassium dihydrogen phosphate (KH_2_PO_4_), nickel nitrate (NiNO_3_), hydrogen peroxide (H_2_O_2_), sodium chloride (NaCl),potassium permanganate (KMnO_4_), potassium chloride (KCl), hydrochloric acid (HCl), nitric acid (HNO_3_), and sulfuric acid (H_2_SO_4_) were purchased from Merck (Germany). The healthy human blood samples were collected from a clinical diagnostic laboratory, where *Mycobacterium* infection was already checked by culture plate method. The samples were declared (certified) free from any disease including *Mycobacterium* infection by the diagnostic laboratory. Zero-grade hydrogen (H_2_) and nitrogen (N_2_) gases were purchased from Sigma Gases (India). All solutions were prepared in Type 1 ultrapure water using the Elix Mili Q system (USA).

### Synthesis of GO

The material was prepared from graphite powder by Hummers’ method with some modifications (34, 35). Briefly, 1 g of graphite powder, 0.75 g of sodium nitrate, and 37.5 mL of concentrated H_2_SO_4_ (98% w/w) were transferred to a 1 L conical flask. The flask was placed on an ice bath under stirred conditions using a magnetic stirrer. Approximately 4.5 g of KMnO_4_ was added slowly to the mixture. The mixture turned dark green. The solution was stirred for 2 h and then removed from the ice bath, and left at ~30°C under stirring for five days. The solution became viscous and turned dark brown. Approximately 100 mL of H_2_SO_4_ (5% w/w) was added to the solution. There was no change in color, indicating the formation of GO. The solution was stirred for another 2 h. Approximately 3 mL of 30% (w/w) H_2_O_2_ was added to the solution. The solution turned golden yellow. Stirring continued for 1 h. The solution was centrifuged at 3842 g for 20 min and the suspension was washed five times each with distilled water and 1 M HCl, and finally with distilled water until pH of the suspension reached ~7. The suspension was transferred to Petri dishes and left for drying in air for 4 days.

### Preparation of the NiO-GO Mixture

Approximately 1 g of dried GO was added to a 50 mL volume of 0.4 M NiNO_3_ solution. The mixture was sonicated for 2 h to disperse GO in the salt solution. The solution was centrifuged at 3842 g for 20 min. The suspension was transferred to Petri dishes and left to dry in air for 4 days (36, 37). The prepared NiNO_3_-GO mixture was calcined in a tubular furnace at 450°C for 4 h under N_2_ flow at 200 standard cubic cm per min (sccm) to convert NiNO_3_ to NiO. The NiO-GO mixture was subjected to the H_2-_reduction (200 sccm) at 600°C for 2 h to convert NiO to Ni and GO to rGO. Reduction temperature was determined using temperature-programmed reduction (TPR) analysis.

### Preparation of PANI

Approximately 9 mL of aniline was added to 150 mL of distilled water in a round-bottom flask. The flask was placed on the ice bath. The temperature of the mixture was adjusted between 0 - 5°C. The solution was stirred continuously using a magnetic stirrer. Approximately 10 mL of 9 M HCl was added dropwise to the solution. Approximately 100 mL of (NH_4_)_2_S_2_O_8_ (11% w/w) solution was added dropwise to the aniline solution. The solution turned dark green. The solution was stirred for another 2 h and then centrifuged at 3842 g for 10 min. The supernatant was discarded and the residual suspension of PANI was washed with distilled water until its pH reached ~7 (38).

### Synthesis of the Ni-rGO-PANI Electrode

The prepared Ni-rGO mixture (~4% w/w) was added to PANI. The mixture was sonicated for 2 h in distilled water to uniformly disperse Ni-rGO in PANI. The sonicated mixture was centrifuged at 3842 g for 15 min. The residual slurry suspension was cast as a ~2 mm-thick film on Petri dishes and left to dry in air for 5 days. The dried Ni-rGO-PANI metal-carbon-polymer composite film was cut into rectangular (10 mm x 5 mm) pieces to be used as the test electrodes. Samples of rGO-PANI (without Ni) and the PANI substrate (without Ni and rGO) were prepared for comparison.

### Preparation of PBS Buffer and Synthetic Clinical Samples

Approximately 8 g of NaCl, 0.2 g of KCl, 1.44 g of Na_2_HPO_4_, and 0.24 g of KH_2_PO_4_were transferred to 800 mL of Milli-Q water in a 1 L-conical flask. The solution pH was adjusted to 7.2 using 1 M HCl, and the final volume was maintained at 1 L. The solution was sterilized for 15 min in an autoclave at 121°C. Approximately 1 mL of the PBS was mixed in ESAT-6 protein to prepare the stock solution of 1 µg mL^-1^. Stock solutions of the interfering biomolecules (Ag85, AP, Glu, U, AA, Cre, Chl, BA, and Glt) were also prepared in the same PBS solution.

Blood samples (2 ml) were collected in EDTA tube containing anticoagulant enzymes and stored at 4°C for the clinical measurements. The as-received samples were centrifuged at 2400 rpm for 15 min to separate the erythrocytes (RBC and WBC) from blood plasma (supernatant fluid). Approximately 0.01 mL plasma solution was used to prepare the synthetic clinical samples at different concentrations of ESAT-6.

### Electrode Surface Modification

**Figure 1:**
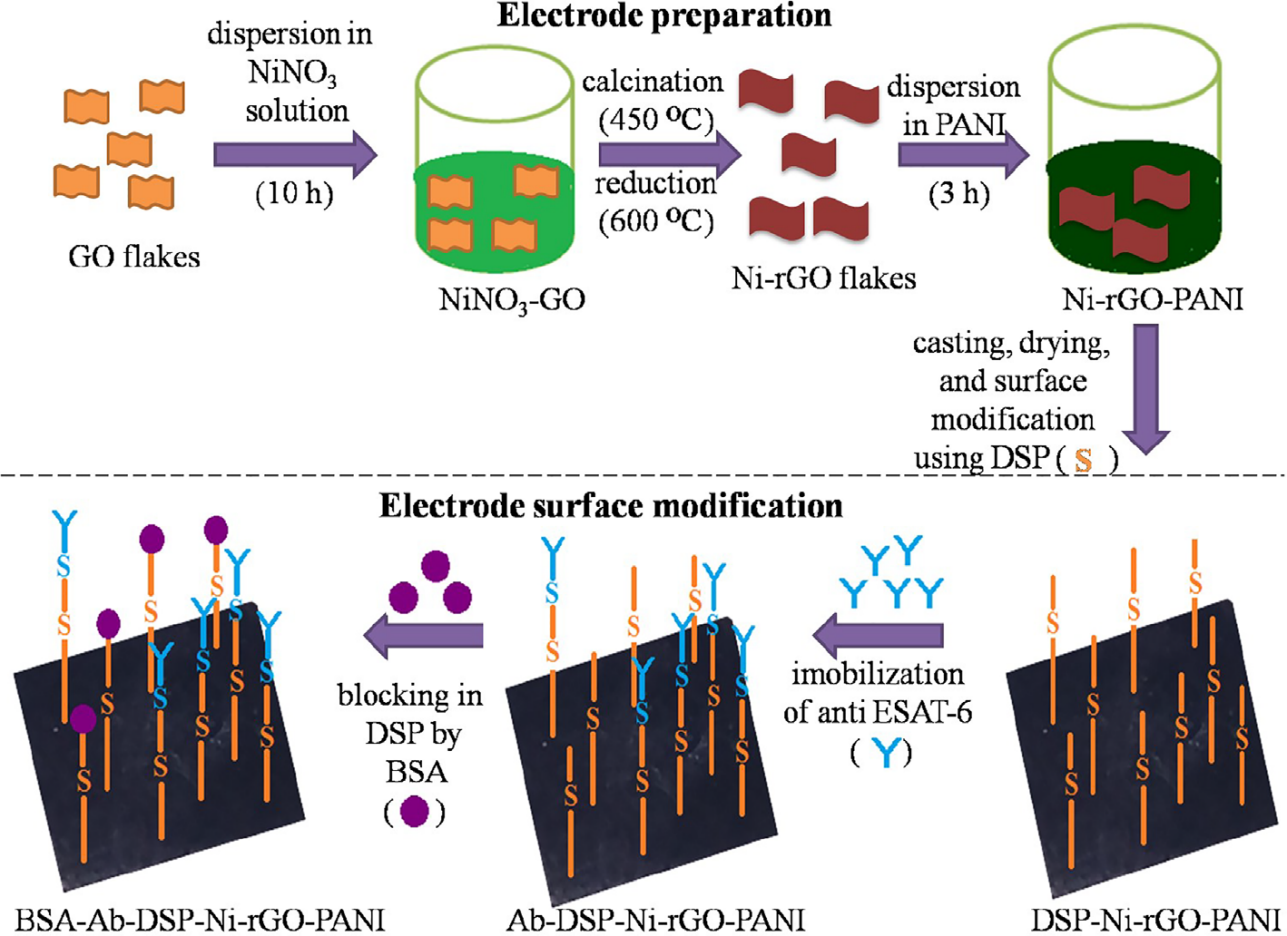
Electrode preparation and surface modification.

The prepared Ni-rGO-PANI electrodes were first washed with distilled water and dried in N_2_ flow. Dried electrodes were immersed in an acetone solution containing 1 mM of DSP for 1 h at ~30°C. DSP interacts with the Ni NPs through Ni-sulfide bond. At the other end, it interacts with the antibody *via* amide bond (17, 39, 40). The DSP-coated electrodes were washed with acetone and distilled water to remove excess DSP from the electrode surface. Washed electrodes (DSP-Ni-rGO-PANI) were immersed in PBS buffer (pH 7.2) containing 100 ng mL^-1^ of anti-ESAT-6 antibody. The entire electrode and antibody solution were incubated overnight at 4°C. The incubated electrode samples were washed using the PBS buffer to remove unbound antibodies. The washed samples (Ab-DSP-Ni-rGO-PANI) were soaked in BSA (1% w/w) solution at ~30°C for 1 h to block the free sites in DSP. The samples were washed with PBS to remove excess BSA. The prepared material (BSA-Ab-DSP-Ni-rGO-PANI) was used as the sensing electrode for ESAT-6. In the surface modification step described above, DSP served as the cross-linking agent for the anti-ESAT-6 antibody. DSP contains two amine-reactive N-hydroxyl succinimide (NHS) esters that react with primary amines on the antibody to make a stable amide bond with subsequent release of the NHS group. **Figure 1** describes the preparation- and surface modification steps for the sensing electrode schematically (17, 39, 40).

### Physicochemical Characterization

Ni loading in the electrode material was determined by leaching the metal from an approximately 0.1 g of the Ni-rGO sample in 10 mL of concentrated HNO_3_. The mixture was heated at 80°C for 2 days until the solution became colorless. After cooling to ~30°C, the solution turned light green. Solution volume was maintained at 10 mL using 1% (w/w) nitric acid. Metal concentration in the leachate was measured using atomic absorption spectrometry (AAS) (Varian AA-420, USA) equipped with a deuterium background corrector and a hollow cathode lamp as the radiation source.

Specific surface area (S_BET_) of the prepared materials was determined using an Autosorb-1C instrument (Quantachrome, USA). N_2_ was used as an absorbate probe molecule at 77 K over the P/P_0_ values ranging from 0.01 to 0.99. The reduction temperature of NiO-GO was determined from TPR analysis using the Quantachrome instrument. H_2_-reduction was performed from 0 to 900°C. A ramp rate of 10°C per min was used for the reduction step. Surfaces of the materials were observed using high-resolution field emission scanning electron microscopy (FE-SEM) (JSM 7100F/JEOL, Netherlands) and the metal distributions were determined using energy-dispersive X-ray spectrometer (EDX) attached to the FE-SEM. Crystal lattices of the materials were determined using X-ray diffraction spectroscopy (XRD) (Pananalytical X’Pert Pro, UK). The samples were dried in vacuum and analysis was performed using Ni-k_α_ radiation (k = 1.54178 A°) in the 2θ range 20–100° at a scan rate of 3° per min. Functional groups in the material surface were determined using Fourier-transform infrared spectroscopy (FT-IR) (Bruker Tensor 27, Germany). Spectra were recorded over the range 600–4000 cm^-1^. Graphitic content in the material was determined using Raman spectroscopy (Spex 1403, Singapore) with a He-Ne laser. The spectra were recorded using 532 nm excitation wavelength over the range 1000–3000 cm^-1^.

### Electrode Electrochemical Characterization

All electrochemical measurements were performed using the Autolab workstation (Metrohm, USA). In the three-electrode assembly, Ni-rGO-PANI was used as the working electrode, and Ag/AgCl and a Pt rod were used as the reference and counter electrodes, respectively. Analysis was performed in PBS solution at pH 7.2; this value was chosen because blood and sputum pH values for patients infected with TB are in the range (7.0– 7.4) (41) and (6.8–7.5) (42), respectively. CV analysis was performed to determine electrode activity towards the antibody-antigen interactions from -1 to 1 V with a starting potential of 0 V at a scan rate of 10 mV s^-1^. EIS measurements were performed to determine impedance on the material surface. The measurements were taken over the frequency range 10^-4^–10^2^ kHz under a set potential of 0 V, integration time of 0.125 s, and amplitude of 0.01 mV. All measurements were performed in triplicate; the data are reported as the mean of the measurements.

## Result and Discussion

### SEM and EDX Analysis

**Figure 2:**
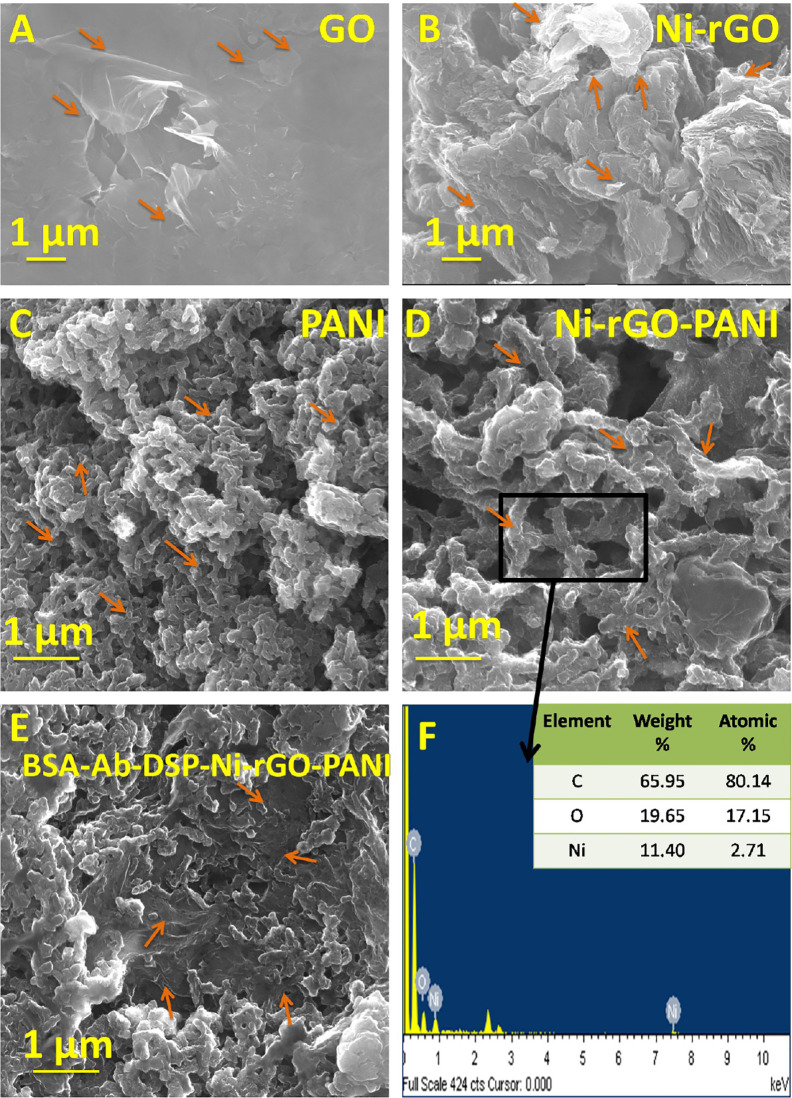
SEM images of **(A)** GO, **(B)** Ni-rGO, **(C)** PANI, **(D)** Ni-rGO-PANI, **(E)** BSA-Ab-DSP-Ni-rGO-PANI, and **(F)** EDX spectra of Ni-rGO-PANI. The arrows indicate the specific structures of the material.

The SEM images of BSA-Ab-DSP-Ni-rGO-PANI at various stages of preparation are shown in **Figures 2A–F**. Flake-like structure was observed in the GO sample (**Figure 2A**). Shiny dots in **Figure 2B** indicate that the Ni NPs are dispersed on the surface of the rGO flakes (indicated by arrow). The presence of Ni NPs on the surface of rGO was confirmed by the EDX analysis. The SEM image of PANI indicated an amorphous structure containing the randomly distributed micron-sized long particles (**Figure 2C**). A relatively more compact and dense structure was observed after the addition of Ni and rGO (**Figure 2D**), confirming the inclusion of rGO as well as Ni NPs. The supplementary file can be referred for the SEM images of the treated samples, viz., SEM images post-surface modification of Ni-rGO-PANI with DSP (**Supplementary Figure 1A**), antibody immobilization (**Supplementary Figure 1B**), and BSA (**Figure 2E** and **Supplementary Figure 1C**). Surface modification of Ni-rGO-PANI with DSP resulted in the coverage of the electrode surface with DSP layer, possibly blocking the pore-mouths, confirmed by the BET surface area analysis. **Figure 2F** and **Supplementary Figures 1D–F** show the EDX spectra of the fresh Ni-rGO-PANI sample and that of the sample after each surface modification step. The inset table shows elemental distributions, which confirms the presence of Ni (11.40% w/w) in Ni-rGO-PANI (before surface modification). The amount successively decreased to 6.4, 5.5 and 3.2% (w/w) after the surface modifications with DSP, Ab and BSA, respectively, confirming the coating of the surface modifying agents.

### Ni-Loading

The amount of Ni in Ni-rGO was quantitatively analyzed by the AAS analysis, and the metal loading was determined to be approximately 59 mg/g. The data indicate a good amount of Ni-loading in the precursor material for the electrode. A relatively higher Ni-loading renders the resulting composite material to be a good electroconductive, which is beneficial for sensing application (28).

### TPR Analysis

**Figure 3:**
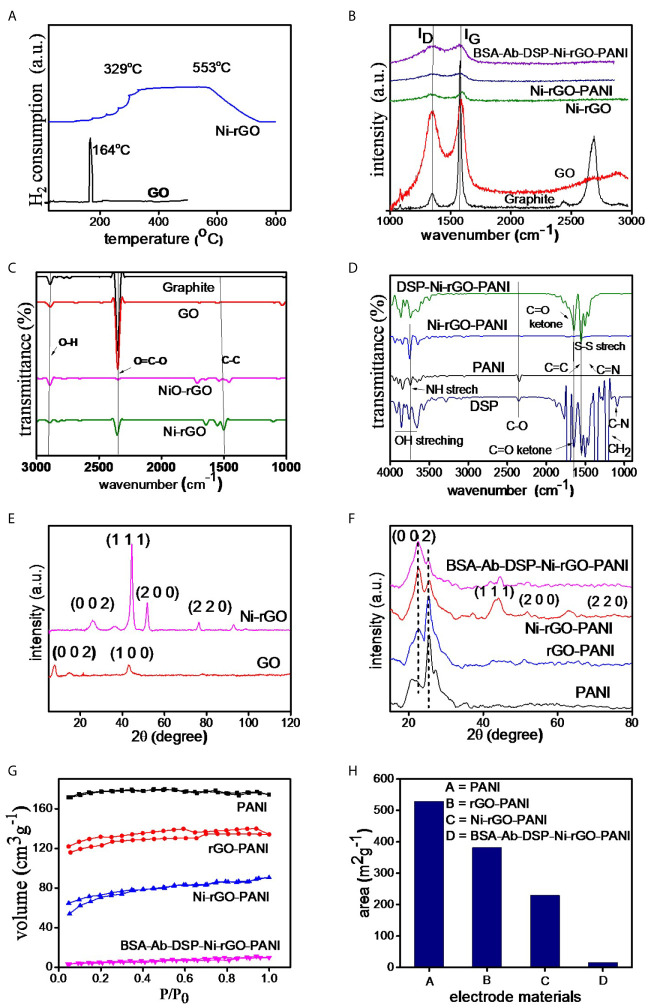
**(A)** TPR of GO and NiO-GO, **(B)** RAMAN spectra of electrode materials, **(C, D)** FT-IR and **(E, F)** XRD spectra of the substrate and surface-modified materials, **(G)** BET isotherms and **(H)** surface area of the materials.

**Figure 3A** shows the TPR spectra of GO and NiO-rGO. A sharp peak is observed at ~164°C in GO, indicating the reduction of GO below 200°C. Only one peak was observed for NiO-rGO over the temperature range 329 – 554°C, indicating the reduction of NiO to Ni. No peak was observed for GO, confirming the formation of rGO (reduction of GO to rGO) during calcinations. As mentioned earlier, the H_2_-reduction was performed at 600°C to convert NiO to Ni in the NiO-rGO mixture.

### Raman Analysis

Raman spectra of the prepared materials (graphite powder, GO, Ni-rGO, Ni-rGO-PANI, and BSA-Ab-DSP-Ni-rGO-PANI) are shown in **Figure 3B**. Two bands, namely D and G, were detected in all materials; these are attributed to the D-band (~1343.27 cm^-1^) signifying the disordered phase, and the G-band (~1589.43 cm^-1^) signifying the graphitic characteristics of the material. Graphite powder showed an additional 2D band, indicating the layered structure of the material. The I_D_/I_G_ ratios were 0.10, 0.89, 0.98, 0.97, and 0.99 for graphite, GO, Ni-rGO, Ni-rGO-PANI, and BSA-Ab-DSP-Ni-rGO-PANI, respectively. An increase in the ratio is observed for Ni-rGO, indicating a decrease in the relative graphitic content of the material. Further modification with PANI and antibody did not alter the graphitic characteristics of the material.

### FT-IR Analysis

**Figure 3C** shows the FT-IR spectra of graphitic powder, GO, NiO-rGO, and Ni-rGO. Common characteristic peaks observed at ~1200, 2300, and 2800 cm^-1^ in the materials are assigned to the aromatic ring C-C groups, carboxylic O=C-O, and O-H groups, respectively. The intensity of the O=C-O peak decreased in the H_2_-reduced samples, indicating reduction of NiO and GO to Ni and rGO, respectively. **Figure 3D** shows the spectra of the DSP-modified materials. Three characteristic peaks were observed in PANI. Peaks at ~2200, 1750, and 1600 cm^-1^ are attributed to the C-O, C≡N, and C=C and groups, respectively. These peaks were observed in all three PANI-based materials. Five characteristic peaks were observed in DSP. Peaks at ~1750, 1650, 1500, 1250 and 1200 cm^-1^ are attributed to C=C, C=N, S-S, C=O ketone, and H-C-H stretching, respectively. These peaks were also observed in all DSP-coated materials, confirming the DSP coating on Ni-rGO-PANI.

### XRD Analysis

**Figure 3E** shows the XRD spectra of GO and Ni-rGO. The peak located at 2θ angle ~10° in GO corresponds to the (0 0 1) crystallographic plane of C. However, the peak is absent in the rGO-containing materials, indicating conversion of GO to rGO during the H_2_-reduction step. The characteristic peaks observed at ~35, 43, and 78° in Ni-rGO correspond to the crystallographic (0 0 2), (1 1 1), and (2 2 0) planes of Ni, respectively. The peak at ~30° in Ni-rGO-PANI is attributed to the presence of PANI and those at ~50, 55, and 75° are attributed to the (1 1 1), (2 0 0), and (2 2 0) planes of Ni, confirmed from JCPDS#70-0989 (**Figure 3F**).

### BET Analysis

**Figure 3G** shows N_2_ adsorption-desorption isotherms of the prepared materials. The isotherms show the type-II characteristics as per the IUPAC classifications. BET areas were calculated from the isotherms. A relatively higher surface area was measured in the PANI film, indicating the porous characteristics of the material; this is consistent with SEM results that revealed pores in the material, discussed earlier (**Figure 3H**). BET surface area decreased as expected, with inclusion of rGO and Ni NPs in PANI, as the pores were partially blocked. It may be mentioned that the main objective of performing the BET analysis was to determine the surface area of the substrate (PANI). Coating (surface modification) of the electrode with DSP/Ab/BSA caused blocking of the pore mouths on the electrode surface. Thus, the BET area significantly decreased, corroborating the formation of a DSP/Ab/BSA layer on the Ni-rGO-PANI surface (**Supplementary Table 1**).

### CV and EIS Analysis

CV was performed on PANI, rGO-PANI, and Ni-rGO-PANI. Prior to the analysis, the scan rate was optimized using CV analysis at different rates 10, 25, 50, 75, 100, 125, and 150 mV s^-1^) (**Supplementary Figure 2A**). The current responses of the bare electrode (without surface modifications) in PBS showed a liner relationship with the square root of the scan rates (**Supplementary Figure 2B**), indicating the diffusion-controlled electron exchange mechanism at the surface of the electrode (43, 44).

**Figure 4:**
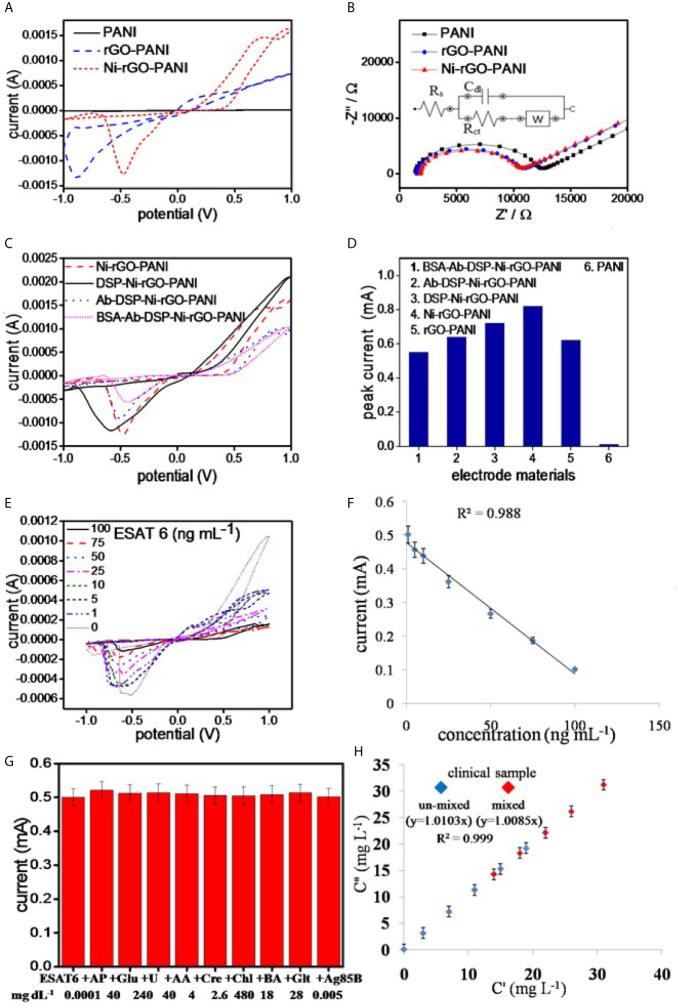
**(A)** CV and **(B)** EIS analysis of the electrode materials, **(C)** CV analysis of the surface modified electrodes, **(D)** peak currents in the surface modified materials, **(E)** CV analysis of BSA-Ab-DSP-Ni-rGO-PANI at different ESAT-6 concentration, **(F)** calibration plot for the prepared TB sensor, **(G)** selectivity data measured at LOD, and **(H)** measurements in the synthetic clinical samples (C”) *vs*. prepared ESAT-6 (C’) in the clinical samples.

The best electrode response (sharp and distinguished peak) was determined at 10 mVs^-1^. Hence, all analysis were performed at the scan rate of 10 mVs^-1^. PANI showed negligible peak current during the CV measurements in PBS alone, as also reported in the literature (**Figure 4A**) (45, 46). However, rGO-PANI showed a reduction peak at the potential of approximately -0.1 V with a current response of 0.62 mA, attributed to the electrocatalytic effects of rGO (47–49). The reduction potential slightly shifted in the range 0.5 – 0.6 V in Ni-rGO-PANI. However, a relatively higher current response (0.82 mA) was measured, attributed to the inclusion of the electrocatalytic Ni NPs in the material (50–52). EIS measurements were performed to determine the surface impedances of PANI, rGO-PANI, and Ni-rGO-PANI (**Figure 4B**). Data were fitted with Randles model circuit, as shown in the inset of the figure. **Supplementary Figure 2C** shows the magnified fitting-segment over the low impedance values for clarity. Solution resistances (Rs) were approximately the same (~1230 Ω) for all materials. Charge-transfer resistance (Rct) was approximately 11080 Ω at the PANI surface. Rct decreased to ~10450 and 8804 Ω in rGO-PANI and Ni-rGO-PANI, respectively, which indicates low impedance on Ni-rGO-PANI; this is attributed to increased mobility of free electrons at the electrode surface because of the synergistic effects between rGO and Ni NPs (20, 45, 46, 53). Based on the data, PANI and rGO-PANI were removed from further consideration; in this study, only the Ni-rGO-PANI surface was modified with the antibody for *Mtb* sensing.

CV analysis of DSP-coated Ni-rGO-PANI displayed a similar reduction peak to that of Ni-rGO-PANI, however, at a diminished reduction potential (0.6 V) and peak current (0.52 mA) (**Figure 4C**). Further modifications of DSP-Ni-rGO-PANI by the antibody and BSA did not result in decrease of the reduction potential. The responses diminished in the surface-modified materials (**Figure 4C**), as the relative amounts of Ni in the surface-modified electrodes decreased, confirmed by the EDX analysis (**Supplementary Figure 1**). The role of the Ni NPs during sensing is, therefore, only to enhance the electroconductivity of the electrode. **Figure 4D** summarizes the peak currents measured for the materials.

### Sensor Calibration

The prepared BSA-Ab-DSP-Ni-rGO-PANI sensor was calibrated using seven different concentrations of the biomarker over the range 1 – 100 ng mL^-1^. Peak currents were measured over the range -0.5 – -0.6 V during CV analysis (**Figure 4E**). At 0 ng/mL, the antibodies (bounded at the electrode surface) were free without adsorbing any protein molecules. Over the concentration range 1 – 100 ng mL^-1^, the antibodies interacted with the protein molecules *via* adsorption. The resistivity of the electrode increased, which caused decrease in the activity of electrode, requiring a relatively higher potential for the response. Therefore, the peak position slightly shifted to the more negative potentials. Currents decreased with increasing concentrations; this is attributed to an increased resistance of the electrode materials resulting from increased ESAT-6 bonding to the antibody (anti-ESAT-6) at high concentrations. When a protein molecule binds to an antibody, the insulating property of the electrode surface increases because of the non-conductive characteristics of the protein molecule. The insulation on the surface of the electrode blocks the movement of electrons, resulting in decrease of the peek currents during the CV analysis (17). Calibration data showed a good linearity (R^2^ = 0.988) over the measured ESAT-6 concentration range (**Figure 4F**). Current peaks were detected to a minimum concentration of 1 ng mL^-1^. Peak currents corresponding to the measured concentrations, standard deviations (S.D.), and % relative standard deviations (RSD) are presented in **Supplementary Table 2**. The limit of detection (LOD) and limit of quantification (LOQ) for ESAT-6 are 1.042 and 3.065 ng mL^-1^, respectively, calculated using the following equations:

$$\text{LOD}=3\times \sigma_{\text{blank}}/\text{S}$$

LOQ=10×σ/S.

where, σ_blank_, σ, and S are the standard deviation of the blank electrode, the standard deviation of the lowest concentration measured during the calibration, and the slope of calibration line, respectively.

### Selectivity and Interference Tests

The prepared sensor was tested for ESAT-6 along with another biomolecule present in human blood of healthy and infected people to confirm the selectivity of the sensor towards ESAT-6 protein molecule. Ag85B is also a TB biomarker, which is a secretary protein of *Mycobacterium tuberculosis.* Similarly, the biomolecules such as AP, Glu, U, AA, Cre, Chl, BA, and Glt are commonly present in blood and may interfere with the measurements. The concentrations of these molecules were used at the concentration levels two-times the respective upper permissible concentration in human blood (22, 54–56). The measurements were performed over the potential range optimized earlier. The ESAT-6 concentrations were taken at the lower and upper concentrations (1 and 100 ng mL^-1^, respectively) of the calibration curve. The CV measurements revealed no peaks other than that for ESAT-6 in the presence of the biomolecules (**Supplementary Figure 2D**). Also, the peak current value was approximately the same as before (without biomolecules) at LOD (**Figure 4G**) and at high concentration of the calibration plot (**Supplementary Figure 2E**). The data, therefore, clearly indicated the selectively of the prepared sensor in this study towards ESAT-6 with negligible interference of the other biomolecules towards the detection of ESAT-6.

### Measurements in Blood Samples

The clinical blood samples were processed as described earlier in the Materials and Method section. The ESAT-6 protein was mixed at five different concentrations in blood plasma, and the concentration were measured using the CV analysis at the earlier optimized parameters. **Supplementary Table 3** shows the SD values less than 0.2 and the RSD values less than 2%, clearly indicating the BSA-Ab-DSP-Ni-rGO-PANI electrode capable of measuring the ESAT-6 concentrations accurately in human blood. To validate the clinical measurements, five extra blood samples were prepared by mixing two clinical samples in the same volume (1:1). The new samples were termed as AC (A+C), AD (A+D), BD (B+D), CD (C+D) and CE (C+E). The CV measurements were taken and the data for peak currents are presented in **Figure 4H**. The SD and RSD values were determined to be < 1and 2%, respectively. A calibration curve was plotted against the actual *vs*. prepared concentrations. The regressed lines were found to be approximately linear for the un-mixed and mixed samples. The data confirm that the BSA-Ab-DSP-Ni-rGO-PANI electrode was capable of detecting ESAT-6 in human blood efficiently and accurately.

### Measurement in Healthy (Recovered From Tuberculosis Infection) Blood Samples

ESAT-6 concentrations were measured in the blood samples of the patients recovered from the tuberculosis infection. As mentioned earlier, the samples were collected from the clinical diagnostic laboratory. Blood cells were separated as per the previously described procedure. As shown in the CV spectra, the peak intensity was measured to be the same as that for 0 ng/ml concentration of the biomarker (ESAT-6) (**Supplementary Figure 2F**). It was inferred that ESAT-6 in healthy people was absent or below the detection limit of the sensor.

### Reuse of Spent Electrodes

Spent electrodes (BSA-Ab-DSP-Ni-rGO-PANI) were repeatedly washed with acetone and PBS, and dried in a N_2_ atmosphere. Washed electrodes were subjected to another surface modification using the DSP cross-linker and ESAT-6 antibody. CV data were recorded at each step of the modification, as with the earlier electrodes. Sensor responses of the reused electrodes were approximately the same as those of the fresh electrodes (**Supplementary Figure 2G**). The washing, surface modification, and sensing steps were repeated more than ten times with the same electrode; CV data confirmed approximately the same response as before for the BSA-Ab-DSP-Ni-rGO-PANI electrode (**Supplementary Figure 2H**). Clearly, the Ni-rGO-PANI electrode (substrate/base) material can be used multiple times without decrease in its activity towards TB detection.

### Stability of the Electrode

**Figure 5:**
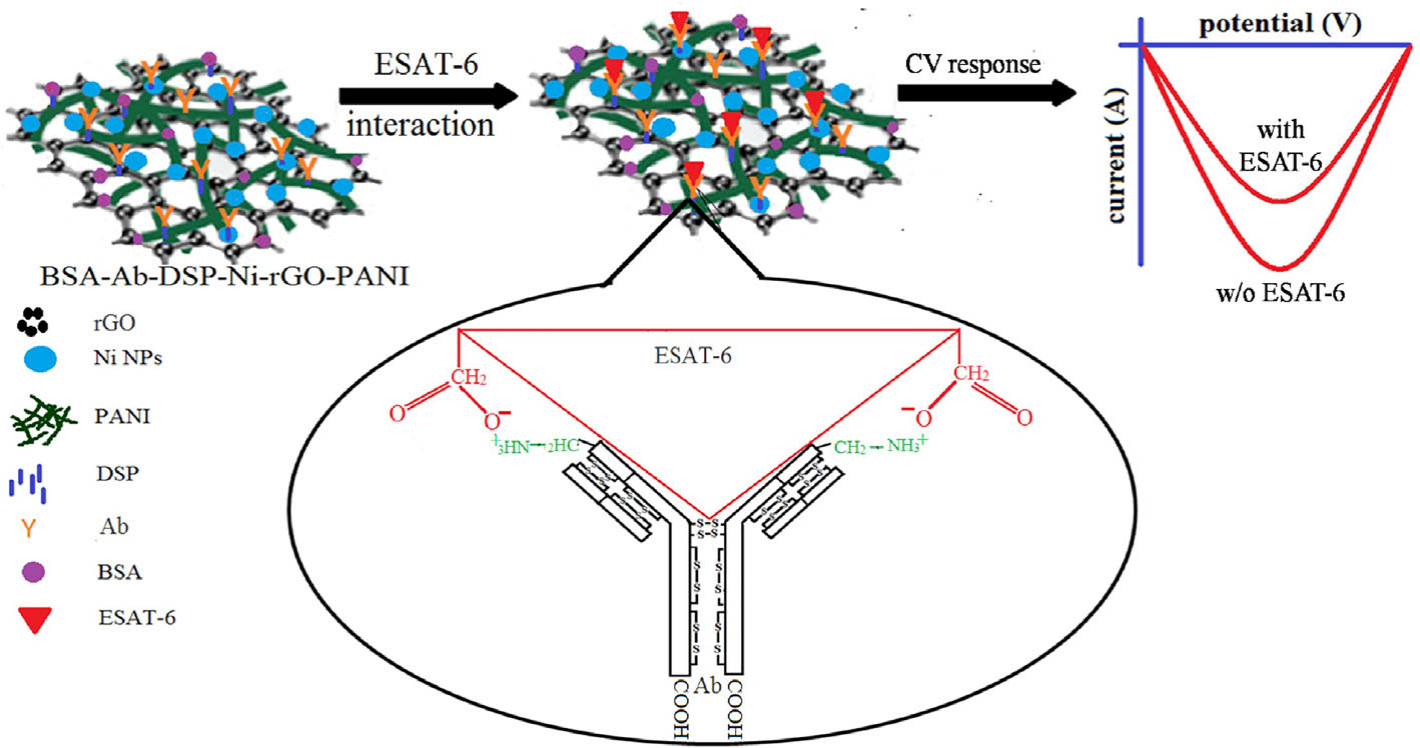
Mechanism of ESAT-6 detection.

BSA-Ab-DSP-Ni-rGO-PANI electrode samples were stored for 6 months in the refrigerator at 0°C under a sterile environment. CV analysis was performed on the preserved electrodes under the same conditions as described earlier to assess the stability of the material. Current peaks had approximately the same magnitudes as measured earlier for the fresh samples over the same potential range, indicating the material to be stable when stored in a sterilized environment (**Supplementary Figure 2I**). A plausible mechanism of the antigen–antibody interaction and ESAT-6 detection is schematically described in **Figure 5**. The ESAT-6 antigen binds with the antibody (anti ESAT-6) through ionic interaction between the O^-^ of carboxylic group in the antigen and ammonium ion $\left(\text{N}{\text{H}}_{3}^{+}\right)$ of peptide chain in antibody *via* the electron transfer (57). During the sensing, the responses decreased with increasing concentrations of ESAT-6, as the antigen formed insulating layers, blocking the electron transfer (**Figure 5**) (17). **Supplementary Figure 3** shows the digital photograph of the prepared electrode (**Supplementary Figure 3A**) and the sensing instrument (**Supplementary Figure 3B**) (58, 59).

We compared, wherever possible, the performances of the sensor prepared in this study with those of the electrochemical sensors discussed in the literature (**Table 1**) (5–7, 9, 12–14, 17, 54, 58). Using different biomarkers and their corresponding recognition elements, most of the *Mtb* sensors displayed a low detection limit and good linearity. In addition, reproducibility among these sensors is reported between 3 and 10 times. As mentioned earlier, only Diouani et al. (2016) have developed the anti-ESAT-6 recognition element-based electrochemical TB biosensor, however, using a relatively expensive SPE (17). On the other hand, the *Mtb* sensor developed in this study is based on the Ni-rGO-PANI electrode and uses the same recognition element (ESAT-6). The LOD of the sensor is 1.042 ng mL^-1^, which is significantly lower than most of the previously developed sensors. Furthermore, the electrode material is stable and reusable, and the assay time is short.

**Table 1:** Comparative performances of the TB electrochemical sensors.

Biomarker	Recognition element	Detection* technique	Detection limit	Stability	Reusability	Reproducibility	Linearity	Linearity range(µg mL^-1^)	Assay time	Ref.
ESAT-6	Anti ESAT-6 (on Ni-rGO-PANI electrode)	CV	1.042 ng mL^-1^	> 6 months	> 10 times	>3 times	0.9814	0.001 - 0.01	~ 15 min	This study
ESAT-6	Anti ESAT-6 antibody (on gold-plated SPE)	SWV	7 ng mL^-1^	**-**	**-**	3 times	0.9920	0.01 - 50	–	17
Mycolic acid	Anti mycolic acid antibody	EIS	**-**	**-**	**-**	**-**	**-**		–	7
*Mtb*	Anti *Mtb* antibodies	CV	1 ng mL^-1^	**-**	**-**	3 times	0.999	0.05 - 0.5	–	6
*Mtb* DNA	DNA probe	DPV	1 CFU mL^-1^	**-**	**-**	**-**	**-**		90 min	9
Interferon γ	DNA aptamer	SWV	1 ng mL^-1^	**-**	**-**	10 times	0.9801	0.005- 0.16	–	54
*Mtb* DNA	DNA Probe	DPV	10 μg mL^-1^	**-**	**-**	–	0.8591	0.01 - 10	–	5
*Mtb* DNA	DNA Probe	CV	50 μg mL^-1^	2-3 weeks	**-**	10 times	0.9870		–	58
E-DNA	DNA Probe	SWV	0.03 fM	–	**-**	4 times	–		–	14
*Mtb* DNA	DNA aptamer	EIS	100 CFU mL^-1^	–	**-**	3 times	0.9375	10^-5^-0.01	2 h	12
*Mtb* DNA	DNA Probe	DVP	1.25 ng mL^-1^	–	**-**	–	–		–	13

*SWV, square wave voltammetry; DPV, differential pulse voltammetry.

## Conclusions

An electroconductive Ni-rGO-PANI electrode-based immunosensor was prepared to quantitatively detect ESAT-6, a biomarker for *Mtb*. Various physicochemical and electrochemical characterization techniques confirmed the effective step-wise surface modifications of the electrode with DSP, antibody, and BSA. CV analysis (potential range -1 to +1 V, starting potential 0 V, scan rate 10 mV s^-1^) demonstrated a decrease in peak current and distinct peak-to-peak separation, indicating the successful immobilization of the antibody recognition element on the electrode surface. The electrode was able to detect ESAT-6 over the concentration range 1 - 100 ng mL^-1^ with good linearity (R^2^ = 0.988). Analysis revealed that Ni-rGO-PANI is stable and that the sensor could be reused repeatedly after surface cleaning and modification; thus, both the material and preparation method are cost-effective. Future tests with the ESAT-6 immunosensor using serum and sputum from both TB-positive and -negative patients are required to implement this assay for a rapid and specific TB diagnosis. The BSA-AB-DSP-Ni-rGO-PANI-based immunosensor developed in this study will also be tested for the diagnosis of real samples of patients infected with tuberculosis infection, in collaboration with a medical college and/or medical research laboratories.

## Data Availability Statement

The original contributions presented in the study are included in the article/**Supplementary Material**. Further inquiries can be directed to the corresponding authors.

## Author Contributions

RO: Methodology, Data curation, Writing original draft. NV: Supervision, Investigation, Writing - review and editing. PA: Supervision, Writing - review and editing. All authors contributed to the article and approved the submitted version.

## Conflict of Interest

The authors declare that the research was conducted in the absence of any commercial or financial relationships that could be construed as a potential conflict of interest.
