# Supplementary material for: Development of ESAT-6 Based Immunosensor for the Detection of Mycobacterium tuberculosis
Source: Front Immunol. 2021 May 19;12:653853. doi: 10.3389/fimmu.2021.653853 (PMC8170314; doi:10.3389/fimmu.2021.653853)
Supplement: Supplementary file 1 [file Table_1.doc]

***Supplementary Material***


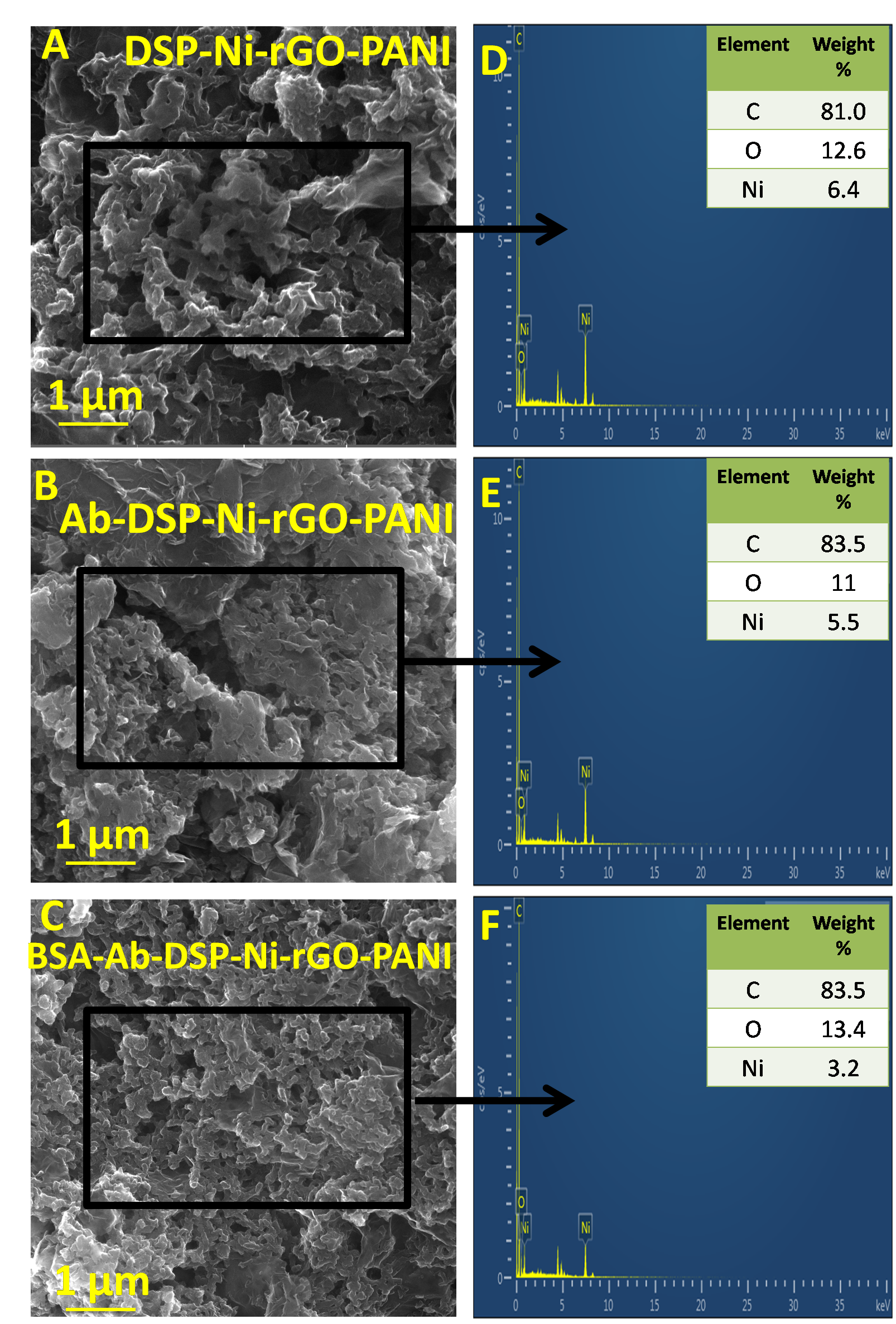


**Supplementary Figure 1.** **(A)** SEM image of the DSP-coated, **(B)** Ab-immobilized and **(C)** BSA-coated electrodes. EDX analysis of **(D)** DSP-coated, **(E)** Ab-immobilized and **(F)** BSA-coated electrodes.


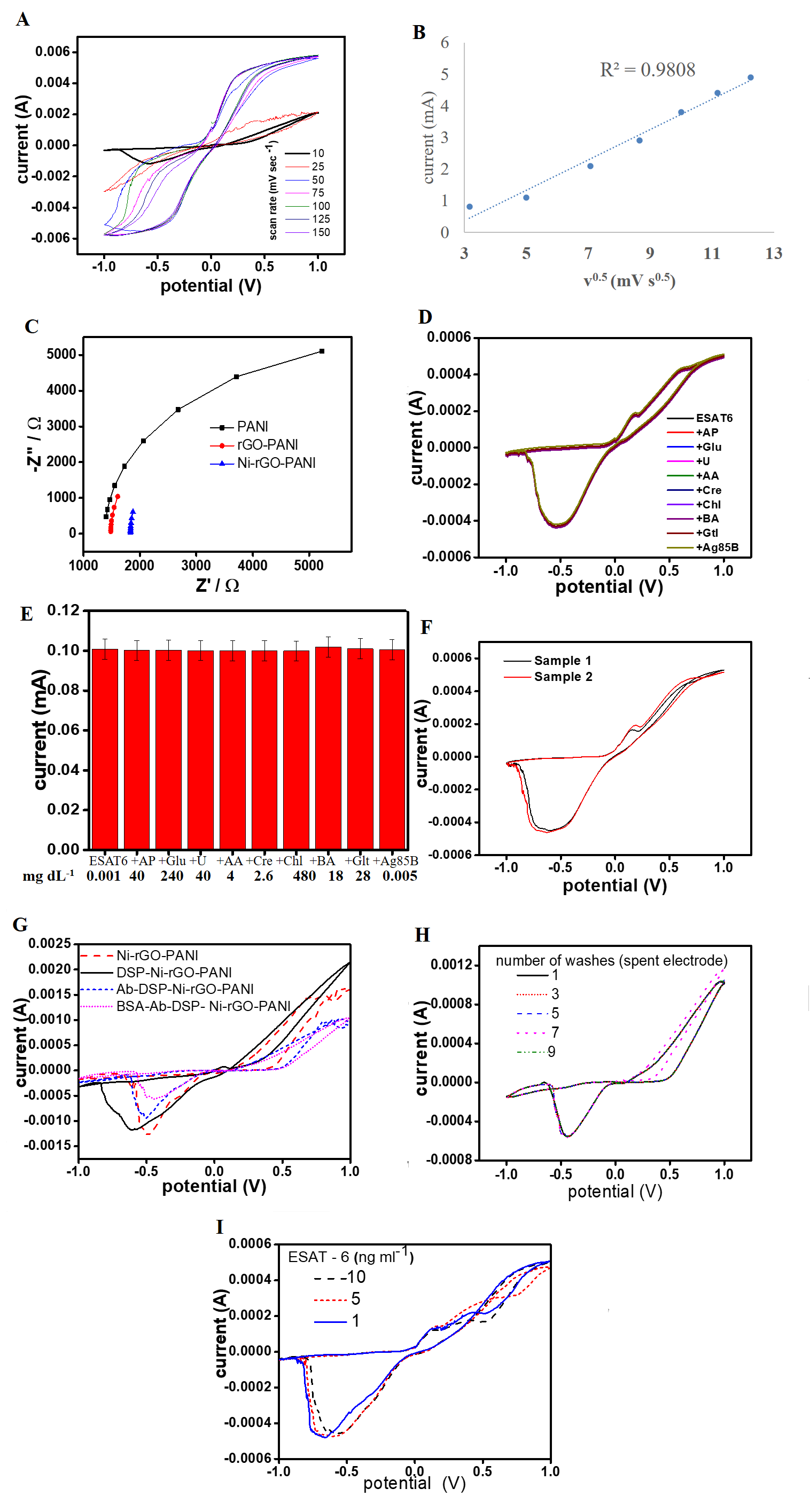


**Supplementary Figure 2.** **(A)** CV analysis for Ni-rGO-PANI at different scan rates, **(B)** peak currents versus *ν*^0.5^ plot for Ni-rGO-PANI **(C)** EIS measurements of the electrode materials over a small impedance-range, **(D)** CV analysis for selectivity at LOD, **(E)** Selectivity on upper concentration of calibration **(F)** CV analysis of healthy blood sample, **(G)** CV analysis of the re-modified (spent) electrode materials, **(H)** CV analysis of the spent electrode up to 10 wash and re-modification, and **(I)** CV data of the BSA-Ab-DSP-Ni-rGO-PANI electrode, post six months-storage.


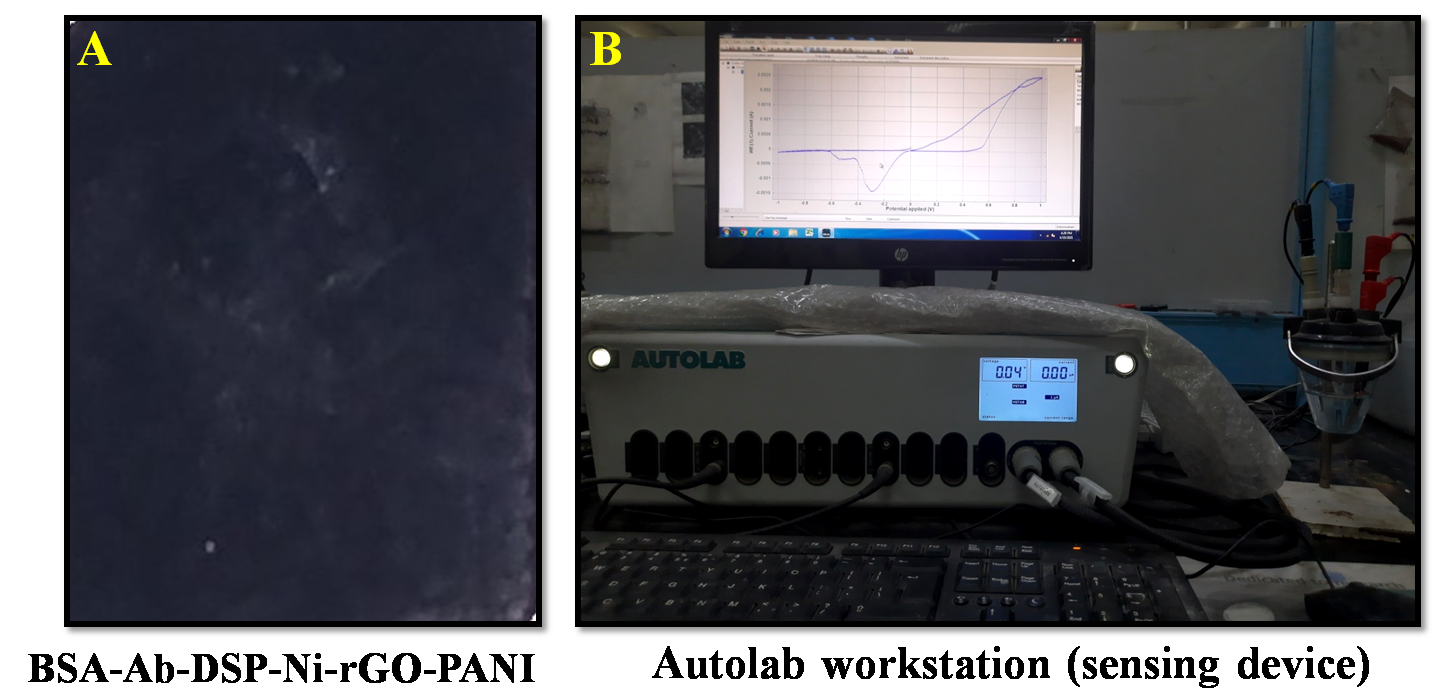


**Supplementary Figure 3.** **(A)** Digital photograph of the prepared electrode and **(B)** sensing instrument.

**Supplementary Table 1.** BET surface areas of the electrode materials after each surface modification step

| **S. No.** | **Materials** | **BET surface area (m^2^/g)** |
| --- | --- | --- |
| **1** | PANI | 530 |
| **2** | rGO-PANI | 383 |
| **3** | Ni-rGO-PANI | 230 |
| **4** | BSA-Ab-DSP-Ni-rGO-PANI | 17 |

**Supplementary Table 2.** Peak currents corresponding to the ESAT-6 concentrations

| **ESAT-6 concentrations**  **(ng mL^-1^)** | **Currents**  **(mA) ± S.D.** | **% RSD** |
| --- | --- | --- |
| 100 | 1.007 ± 0.003 | 0.358 |
| 75 | 1.863 ± 0.029 | 1.572 |
| 50 | 2.6 ± 0.065 | 2.522 |
| 25 | 3.561 ± 0.0569 | 1.599 |
| 10 | 4.332 ± 0.049 | 1.134 |
| 5 | 4.582 ± 0.025 | 0.561 |
| 1 | 5.016 ± 0.011 | 0.238 |

**Supplementary Table 3.** Measurements for synthetic clinical samples

| **Sample Name** | **Concentration of the clinical samples**  **(mg L^-1^)** | **Concentration measured using prepared electrode (mg L^-1^) ± S.D** | **% RSD** |
| --- | --- | --- | --- |
| **Synthetic** | | | |
| A | 3 | 3.12 ± 0.03 | 0.76 |
| B | 7 | 7.18 ± 0.01 | 0.25 |
| C | 11 | 11.29 ± 0.11 | 2.42 |
| D | 15 | 15.26 ± 0.001 | 0.02 |
| E | 19 | 19.21 ± 0.04 | 1.02 |
| **Mixed (equal volume ratio)** | | | |
| AC (A + C) | 14 | 14.24 ± 0.06 | 1.59 |
| AD (A + D) | 18 | 18.29 ± 0.04 | 1.15 |
| BD (B + D) | 22 | 22.13 ± 0.01 | 0.39 |
| CD (C + D) | 26 | 26.17 ± 0.03 | 0.35 |
| CE (C+E) | 31 | 31.21 ± 0.07 | 1.82 |
